# Proposed update to the taxonomy of the genera *Hepacivirus* and *Pegivirus* within the *Flaviviridae* family

**DOI:** 10.1099/jgv.0.000612

**Published:** 2016-11-10

**Authors:** Donald B. Smith, Paul Becher, Jens Bukh, Ernest A. Gould, Gregor Meyers, Thomas Monath, A. Scott Muerhoff, Alexander Pletnev, Rebecca Rico-Hesse, Jack T. Stapleton, Peter Simmonds

**Affiliations:** ^1^​ Centre for Immunity, Infection and Evolution, University of Edinburgh, Scotland, UK; ^2^​ Institute of Virology, University of Veterinary Medicine, Hannover, Germany; ^3^​ Copenhagen Hepatitis C Program (CO-HEP), Department of Infectious Diseases and Clinical Research Centre, Copenhagen University Hospital, Hvidovre, Denmark; ^4^​ Copenhagen Hepatitis C Program (CO-HEP), Department of Immunology and Microbiology, Faculty of Health and Medical Sciences, University of Copenhagen, Denmark; ^5^​ EHESP French School of Public Health, French Institute of Research for Development (IRD), Aix Marseille Université, EPV UMR_D 190 Emergence des Pathologies Virales, Marseille, France; ^6^​ Institut für Immunologie, Friedrich-Loeffler-Institut, Federal Research Institute for Animal Health, Greifswald-Insel Riems, Germany; ^7^​ Hookipa Biotech AG, Vienna, Austria; ^8^​ PaxVax Inc., Menlo Park and Redwood City, CA, USA; ^9^​ Abbott Diagnostics Research and Development, Abbott Park, IL, USA; ^10^​ Laboratory of Infectious Diseases, National Institute of Allergy and Infectious Diseases (NIAID), National Institutes of Health (NIH), Bethesda, MD, USA; ^11^​ Department of Molecular Virology and Microbiology, Baylor College of Medicine, Houston, TX, USA; ^12^​ Department of Pediatrics, Baylor College of Medicine, Houston, TX, USA; ^13^​ Medical Service, Iowa City Veterans Affairs Medical Center, Iowa City, IA, USA; ^14^​ Department of Internal Medicine, University of Iowa, Iowa City, IA, USA; ^15^​ Department of Microbiology, University of Iowa, Iowa City, IA, USA; ^16^​ Nuffield Department of Medicine, University of Oxford, Oxford, UK

**Keywords:** *Flaviviridae*, *Hepacivirus*, *Pegivirus*, hepatitis C virus, taxonomy

## Abstract

Proposals are described for the assignment of recently reported viruses, infecting rodents, bats and other mammalian species, to new species within the *Hepacivirus* and *Pegivirus* genera (family *Flaviviridae*). Assignments into 14 *Hepacivirus* species (*Hepacivirus A–N*) and 11 *Pegivirus* species (*Pegivirus A–K*) are based on phylogenetic relationships and sequence distances between conserved regions extracted from complete coding sequences for members of each proposed taxon. We propose that the species *Hepatitis C virus* is renamed *Hepacivirus C* in order to acknowledge its unique historical position and so as to minimize confusion. Despite the newly documented genetic diversity of hepaciviruses and pegiviruses, members of these genera remain phylogenetically distinct, and differ in hepatotropism and the possession of a basic core protein; pegiviruses in general lack these features. However, other characteristics that were originally used to support their division into separate genera are no longer definitive; there is overlap between the two genera in the type of internal ribosomal entry site and the presence of miR-122 sites in the 5′ UTR, the predicted number of *N*-linked glycosylation sites in the envelope E1 and E2 proteins, the presence of poly U tracts in the 3′ UTR and the propensity of viruses to establish a persistent infection. While all classified hepaciviruses and pegiviruses have mammalian hosts, the recent description of a hepaci-/pegi-like virus from a shark and the likely existence of further homologues in other non-mammalian species indicate that further species or genera remain to be defined in the future.

## Introduction

A recurrent feature of virus taxonomy is that as more information accumulates on the genetic diversity within established virus taxa such as species and genera, the discrete demarcation criteria originally applied to distinguish between them become blurred.

*Hepatitis C virus* (HCV) has been the only named species within the genus *Hepacivirus* since the genus was created in 1996. Considerable diversity exists between different isolates of HCV (Bukh *et al.*, 1993; Simmonds *et al.*, 1993), with 7 genotypes and 84 subtypes currently recognized (Smith *et al.*, 2014) (https://talk.ictvonline.org/ictv_wikis/flaviviridae/w/sg_flavi/56.hcv-classification). Despite this diversity, all of these viruses are derived from human infections and are associated with acute and chronic liver disease, and there continues to be widespread agreement that they are most appropriately considered as members of a single species. A related virus, GBV-B was identified in 1995 from New World monkeys (Simons *et al.*, 1995a) and is associated with acute liver disease, but remained unclassified. In the last few years, several more divergent viruses have been discovered with genome structures and conserved sequence motifs that are similar to those of HCV and GBV-B and isolated from a variety of host species including dog (Kapoor *et al.*, 2011), horse (Burbelo *et al.*, 2012), bat (Quan *et al.*, 2013), rodent (Drexler *et al.*, 2013; Firth *et al.*, 2014; Kapoor *et al.*, 2013a), Old World monkey (Lauck *et al.*, 2013) and cow (Baechlein *et al.*, 2015; Corman *et al.*, 2015). These viruses differ considerably in their epidemiology and presumed route of transmission from HCV.

Similarly, the *Pegivirus* (pronounced peh-gee virus) genus, when first proposed (Stapleton *et al.*, 2011), comprised two species: *Pegivirus A*, including the viruses GBV-A (Simons *et al.*, 1995a) and GBV-C (Leary *et al.*, 1996; Linnen *et al.*, 1996; Simons *et al.*, 1995b) isolated from primates, and *Pegivirus B*, including viruses derived from bats (Epstein *et al.*, 2010). In the last few years, several papers have described viruses that share many features with *Pegivirus A* and *Pegivirus B*, but which are divergent in genome sequence and structure and infect humans (Berg *et al.*, 2015; Kapoor *et al.*, 2015), bats (Quan *et al.*, 2013), horses (Chandriani *et al.*, 2013; Kapoor *et al.*, 2013b), rodents (Firth *et al.*, 2014; Kapoor *et al.*, 2013a) and pigs (Baechlein *et al.*, 2016). In addition, viruses similar to GBV-C have been discovered in a range of primate species (Birkenmeyer *et al.*, 1998; Sibley *et al.*, 2014).

In the current study, we review the classification of these two genera and have revised the list of features by which the two genera can be distinguished. We additionally describe proposals for the assignment of viruses for which a complete coding sequence is available into a series of species within the two genera and provide demarcation criteria that define these assignments. We propose the creation of 13 additional *Hepacivirus* species and 9 additional *Pegivirus* species.

## Results

### *Hepacivirus* genus

**Fig. 1.**
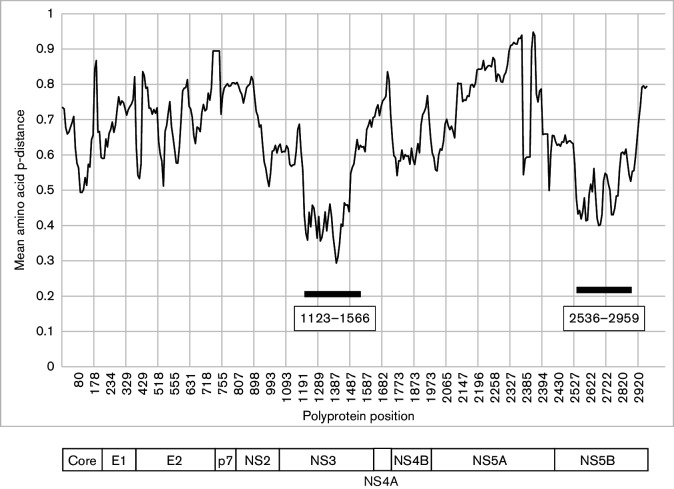
Amino acid divergence across the *Hepacivirus* polyprotein. Mean amino acid p-distances were calculated for 26 aligned *Hepacivirus* polyprotein sequences that differed by >0.1 of amino acid positions using a sliding window of 50 amino acids incremented by 10 residues and plotted against the amino acid position of the start of the fragment. The X-axis scale is uneven because of gaps in the reference sequence (M62321). Two regions with mean p-distances consistently <0.6 are indicated by bars. A schematic representation of the *Hepacivirus* polyprotein is shown to scale below.

**Fig. 2.**
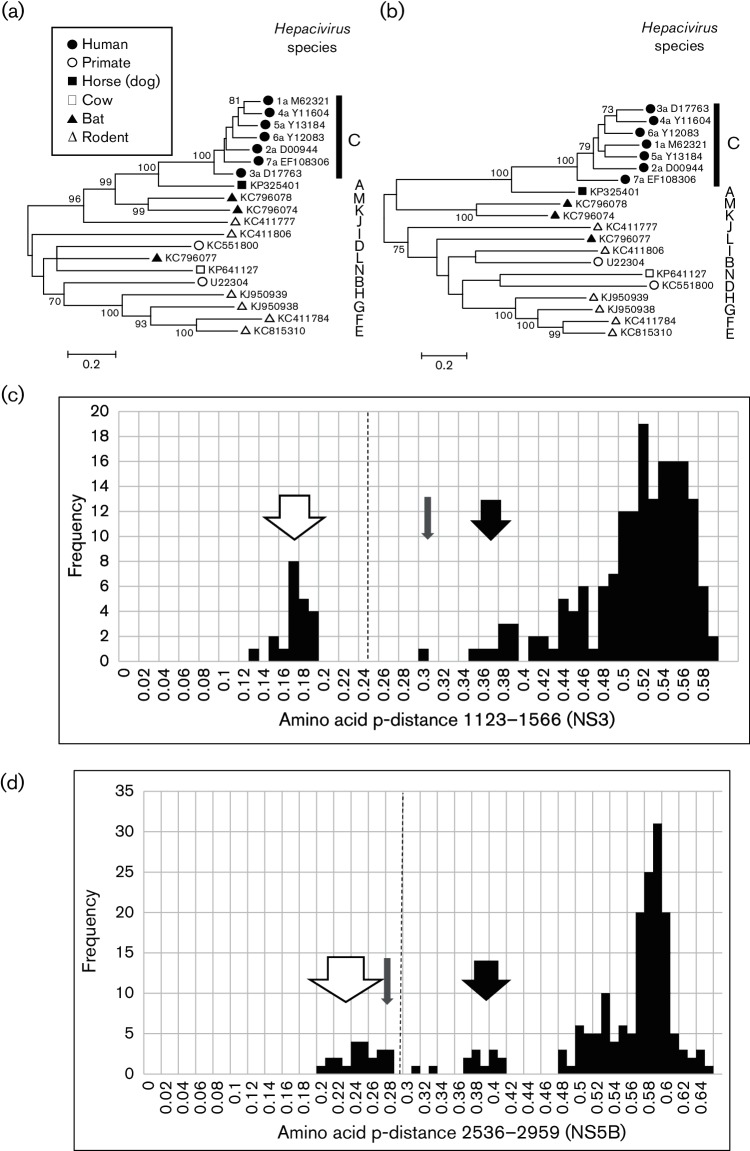
Analysis of *Hepacivirus* conserved regions. Maximum-likelihood trees were produced using mega6 for (a) positions 1123–1566 and (b) 2536–2959 of the virus polyprotein using the Le and Gascuel model and a gamma distribution of variation with invariant sites. Branches observed in >70 % of bootstrap replicates are indicated. Proposed *Hepacivirus* species assignments are indicated by single letters to the right of each branch. (c) Frequency histograms of amino acid p-distance between *Hepacivirus* sequences in the region 1123–1566 and (d) the region 2536–2959. The range of distances between different genotypes of *Hepacivirus C* (HCV) is indicated by an open arrow, between *Hepacivirus G* and *Hepacivirus H* by a shaded arrow and between *Hepacivirus A* and *Hepacivirus C* by a black arrow. The distance that demarcates different species is indicated by a dotted line.

*Hepacivirus* sequences were aligned using muscle and reduced to a set of those differing over their complete coding sequence by amino acid p-distances greater than 0.1. Since different genotypes of HCV all differ by 0.23–0.31, this cut-off would be expected to include all variants likely to represent different species. A scan of mean amino acid p-distance over the coding region revealed two regions where p-distances were consistently less than 0.6: positions 1123–1566 and 2536–2959 (numbered relative to the *Hepacivirus* type species, M62321 (Choo *et al.*, 1989) (Fig. 1), and therefore most informative for phylogenetic comparisons. Phylogenies of *Hepacivirus* sequences in these regions were congruent apart from minor and non-bootstrap-supported rearrangements of deep branches (Fig. 2a, b). For the region 1123–1566, amino acid p-distances were greater than 0.3 apart from distances between different genotypes of HCV, which were 0.12–0.19 (Fig. 2c). A more continuous distribution of amino acid p-distances was observed for the region 2536–2959, with discontinuities centred on distances of 0.35 and 0.45.

Although evidence has been provided for recombination within (González-Candelas *et al.*, 2011) and between *Hepacivirus* species (Thézé *et al.*, 2015), the only known recombinant included in our dataset was the sequence KC796077 (Quan *et al.*, 2013), which is the single known representative of its clade; exclusion of this sequence did not affect the distribution of sequence distances or phylogenetic relationships between the other species (data not shown).

The phylogenetic relationships observed for these two genome regions are consistent with the division of the *Hepacivirus* genus into 14 species which we propose should be named *Hepacivirus A*–*N* (Table 1). Although HCV was the first *Hepacivirus* to be discovered and the type species of its genus, we have chosen to assign it to *Hepacivirus C* rather than *Hepacivirus A* so as to minimize the potential for confusion. To be clear, individual isolates of this virus will still be called hepatitis C virus (HCV), but they will all belong to the species *Hepacivirus C.* Other species are named according to the date of publication of a complete coding sequence, with the exception of *Hepacivirus B* which includes GBV-B (providing a memorable link) and *Hepacivirus A* (canine hepacivirus/non-primate hepacivirus/equine hepacivirus). Demarcation between species is based upon amino acid p-distances of greater than 0.25 in the region 1123–1566 and greater than 0.3 in the region 2536–2959. The rationale for choosing these demarcation points is that they result in HCV and equine hepacivirus isolates being separated into two species, as seems reasonable given their different hosts, while genotypes of HCV remain as members of the same species, reflecting their shared human epidemiology and pathology. The only conflict that arises from these choices is that the rodent-derived sequences KC815310 (Kapoor *et al.*, 2013a) and KC411784 (Drexler *et al.*, 2013) would be considered as two species by comparison of the region 1123–1566 (amino acid p-distance 0.30), but one species by comparison of the region 2536–2959 (amino acid p-distance 0.27). Since these sequences were obtained from different rodent species in the New and Old Worlds, respectively, we prefer a demarcation point that separates these viruses into two species (*Hepacivirus E* and *Hepacivirus F*). The equivocal sequence distances of 0.30 and 0.32 in the region 2536–2959 derive from comparisons between the rodent species *Hepacivirus G* and *Hepacivirus E* and *F*; distances between these species in the region 1123–1566 (0.39, 0.40) are greater than those observed between *Hepacivirus A* and *Hepacivirus C* (0.35–0.38), suggesting that their demarcation into species is appropriate.

**Table 1.** Proposed *Hepacivirus* species

Species	Previous identifier(s)	Type isolate	Accession number	Host/region	Source	5′ UTR	5′ UTR miR-122 sites (CACUCC)	K/R aa before E1	E1/E2 N-X-S/T sites	3′ UTR poly U (nt)	Genome MFED (%)	Tissue tropism	Chronic	Disease
*Hepacivirus A*	Canine hepacivirus Non-primate hepacivirus Equine hepacivirus	NZP1	KP325401	Horse (Dog?)	Serum, liver	IV IRES	1	34	4/9	86	12.8–15.2	Liver	Yes	?
*Hepacivirus B*	GBV-B	T-1053	U22304	New World primate?	Plasma	IV IRES	2	20	2/5	27	8.7	Liver	Rarely	Hepatitis
*Hepacivirus C*	HCV	HCV-1	M62321	Human	Serum, liver	IV IRES	2	31	4/11	10–108‡	7.3–9.9	Liver	Yes	Hepatitis, cirrhosis, cancer
*Hepacivirus D*	Guereza hepacivirus	BWC08	KC551800	Old World primate	Plasma	IV IRES?	1	26	4/4	?	11.6–11.9	?	?	?
*Hepacivirus E*	Rodent hepacivirus	RHV-339	KC815310	New World rodent	Plasma	IV IRES	1	24	2/4	3	4.8–5.1	?	?	?
*Hepacivirus F*	Rodent hepacivirus	NLR07-oct70	KC411784	Old World rodent	Serum	Pegi-like	1	23†	2/3	?	6.8	Liver	?	?
*Hepacivirus G*	Norway rat hepacivirus 1	NrHV-1/NYC-C12	KJ950938	Global rodent	Serum, multiple organs	?	?	22†	3/3	?	5.6	Liver	?	?
*Hepacivirus H*	Norway rat hepacivirus 2	NrHV-2/NYC-E43	KJ950939	Global rodent	Serum, multiple organs	?	?	27†	3/4	?	4.5	Liver	?	?
*Hepacivirus I*	Rodent hepacivirus	SAR-3	KC411806	Old World rodent	Serum	IV IRES	0	29	1/2	5	1.5–1.9	?	?	?
*Hepacivirus J*	Rodent hepacivirus	RMU10-3382	KC411777	Old World rodent	Serum, liver, lung	Pegi-like	2	29	2/7	?	9.8–13.6	Liver	?	?
*Hepacivirus K*	Bat hepacivirus	PDB-829	KC796074	Old World bat	Serum	?	?	34	1/5	?	9.5	?	?	?
*Hepacivirus L*	Bat hepacivirus	PDB-112	KC796077	Old World bat	Serum	?	?	25	2/4	?	7.7	?	?	?
*Hepacivirus M*	Bat hepacivirus	PDB-491.1	KC796078	Old World bat	Serum	?	?	32	1/4	?	9.6–10.8	?	?	?
*Hepacivirus N*	Bovine hepacivirus	463	KP641127	Cow	Serum	IV IRES	1*	25	2/6	4	9.5–10.8	Liver	Yes	?

?, Unknown or uncertain.

*0 in KP641127 but 1 in NC_026797.

†Position of AUG initiation codon uncertain.

‡M62321 has an incomplete 3′ UTR; other *Hepacivirus C* sequences have a run of 10–108 U residues.

According to this schema, the genus *Hepacivirus* contains the species *Hepacivirus A*, including viruses first detected in dogs (canine hepacivirus) (Kapoor *et al.*, 2011), but which subsequently have been detected more frequently in horses (non-primate hepacivirus, equine hepacivirus) (Burbelo *et al.*, 2012). There is much greater virus diversity between equine isolates than is currently described for canine isolates (Pybus & Thézé, 2015), and several studies demonstrate transmission and pathology of infection in the horse (Pfaender *et al.*, 2015; Ramsay *et al.*, 2015; Scheel *et al.*, 2015); these observations are consistent with the horse being the primary host, and for this reason we have used an equine virus (NSP1, KP325401) as the type isolate. *Hepacivirus B* includes GBV-B, a virus initially detected in and capable of infecting New World primates, but that has not been isolated subsequently (Simons *et al.*, 1995a). *Hepacivirus C* includes all currently known genotypes and subtypes of HCV, all of which are confined to humans*. Hepacivirus D* includes sequences derived from colobus monkeys, but about which there is no information for tropism, chronicity or pathogenicity (Lauck *et al.*, 2013). A similar lack of virological or biological information pertains to those species (*Hepacivirus* *E*–*J*) derived from rodents (Drexler *et al.*, 2013; Firth *et al.*, 2014; Kapoor *et al.*, 2013a) and bats (*Hepacivirus K–M*) (Quan *et al.*, 2013). We have retained KC796077 (Quan *et al.*, 2013) as the type species of *Hepacivirus L*, although there is evidence that it is a recombinant (Thézé *et al.*, 2015), since it groups separately from other species whether or not the recombinant region is included (Fig. 2), and since it is the only representative of this clade with a complete coding region sequence. *Hepacivirus N* is represented by viruses isolated from cows and associated with a chronic but asymptomatic liver infection (Baechlein *et al.*, 2015; Corman *et al.*, 2015).

We propose that when the next species of *Hepacivirus* is assigned it should be ‘P’ rather than ‘O’ in order to avoid confusion with the number 0 (zero), and that species beyond X should be named XA, XB, etc., followed by YA, YB, etc. and ZA, ZB, …, ZZ.

### *Pegivirus* genus

**Fig. 3.**
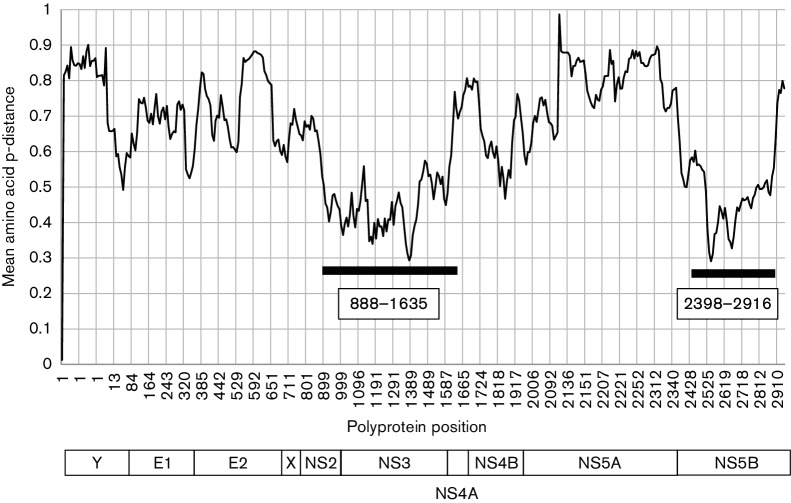
Amino acid divergence across *Pegivirus* polyproteins. Mean amino acid p-distances were calculated for 26 aligned *Pegivirus* polyprotein sequences that differed by >0.11 of amino acid positions using a sliding window of 50 amino acids incremented by 10 residues and plotted against the amino acid position of the start of the fragment. Increments on the X-axis scale are uneven because of unnumbered gaps in the reference sequence (U22303). Two regions with distances consistently <0.6 are indicated by bars. A schematic representation of the *Pegivirus* polyprotein is shown to scale below.

**Fig. 4.**
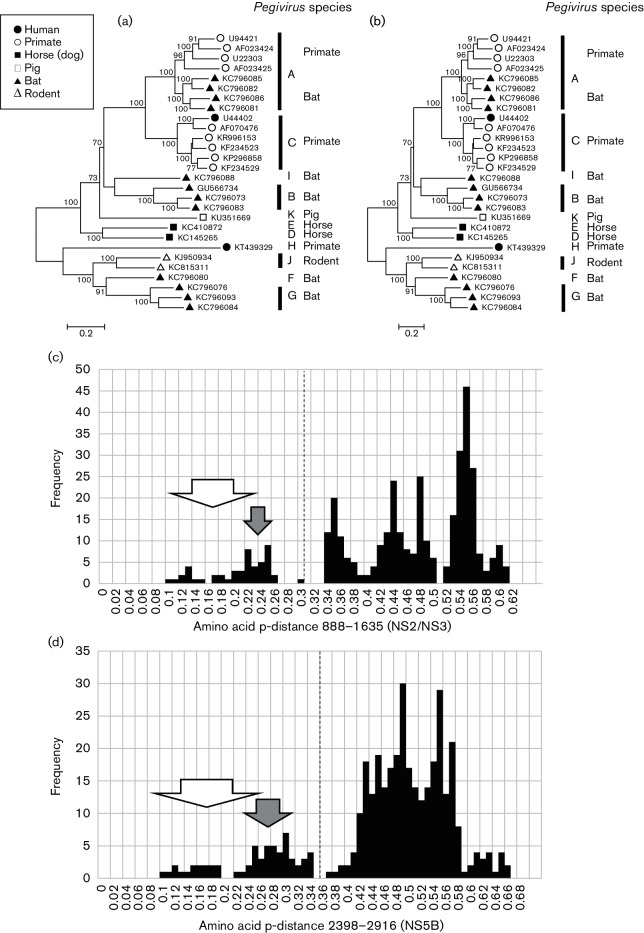
Analysis of *Pegivirus* conserved regions. Maximum-likelihood trees were produced using mega6 for (a) amino acid positions 888–1635 using the Le and Gascuel model with frequencies and a gamma distribution of variation with invariant sites, and for (b) amino acid positions 2398–2916 using the Le and Gascuel model with a gamma distribution of invariant sites. Branches observed in >70 % of bootstrap replicates are indicated. Frequency histograms of amino acid p-distance between *Pegivirus* sequences in the region 888–1635 (c) and the region 2398–2916 (d) Amino acid p-distances between *Pegivirus C* sequences derived from different primate species are indicated by an open arrow, while those between primate- and bat-derived *Pegivirus A* sequences are indicated by a shaded arrow. The distance that demarcates different species is indicated by a broken line.

A set of 26 *Pegivirus* sequences that differed from each other by >0.11 of amino acid positions over their complete coding sequence was used to assess amino acid sequence diversity across the genome. There were two regions where mean amino acid diversity was consistently <0.6: 888–1635 and 2398–2916 (numbered relative to U22303, Fig. 3). Phylogenetic analysis of *Pegivirus* sequences in these two regions produced congruent trees, providing independent evidence that these sequences are phylogenetically distinct (Fig. 4a, b). For both regions, the distribution of amino acid distances between these sequences, whether calculated using sse v1.2 as p-distances, Kimura distances or using a matrix of similarity, was distributed in a series of peaks (Fig. 4c, d) with discontinuities at 0.28–0.34 (positions 888–1635) and 0.35–0.37 (2398–2916). Using an amino acid p-distance of >0.31 for positions 888–1635 to demarcate *Pegivirus* species, the sequences currently described would represent 11 different species (Table 2). These individual species comprise sequences from similar hosts from either the Old or New Worlds with the exception of *Pegivirus A*, which includes sequences derived from New World primates and Old World bats. Two rodent sequences are both included in *Pegivirus I* despite having an ambiguous p-distance for the region 888–1635 (0.303), since they group together on the phylogenetic tree and both are from rodents sampled in the New World. However, if an amino acid p-distance of >0.36 for the region 2398–2916 is used to demarcate species, the amino acid p-distances between *Pegivirus F*, *Pegivirus G* and *Pegivirus J* would all fall below the cut-off. Higher or lower p-distance demarcation points also produce inconsistent assignments. In particular, we could not find demarcation points that divided *Pegivirus A* into exclusively primate or bat-derived groups of sequences.

**Table 2.** Proposed *Pegivirus* species

Species	Previous identifier	Type isolate	Accession number	Host/region	Source	5′ UTR	5′ UTR miR-122 sites (CACUCC)	K/R aa before E1	E1/E2 N-X-S/T sites	3′ UTR poly U (nt)	Genome MFED (%)	Tissue tropism	Chronic	Disease
*Pegivirus A*	GBV-A	T-1053	U22303	New World primate, Old World bat	Plasma	Pegi-like	0	0	1/4	2	10.4–14.1	?	Yes	?
*Pegivirus B*	GBV-D	GBV-D	GU566734	Old World bat	Serum	?	?	5*	1/9	?	9.5	?	?	?
*Pegivirus C*	GBV-C	PNF2161	U44402	Human, Old World primate	Plasma	Pegi-like	0	4	1/3	3	9.4–13.1	Lymphocytes	Yes	No
*Pegivirus D*	Theiler’s disease-associated virus	Horse_A1	KC145265	Horse	Serum	?	0	1	2/7	4	13.1	?	Yes	Serum hepatitis?
*Pegivirus E*	Equine pegivirus	C0035	KC410872	Horse	Serum	Pegi-like	0	8*	1/4	4	11.3	?	Yes	?
*Pegivirus F*	Bat pegivirus	PDB-1698	KC796080	New World bat	Serum	?	1	7*	2/9	?	9.3	?	?	?
*Pegivirus G*	Bat pegivirus	PDB-620	KC796076	Old World bat	Serum	?	?	7*	0/6	?	8.6–12.0	?	?	?
*Pegivirus H*	Human hepegivirus Human pegivirus 2	AK-790	KT439329	Human	Serum	IV IRES	0	5	2/9	3	8.5	?	Yes	?
*Pegivirus I*	Bat pegivirus	PDB-1715	KC796088	New World bat	Serum	?	?	0	1/2	?	9.4	?	?	?
*Pegivirus J*	Rodent pegivirus	CC61	KC815311	New World rodent	Serum, multiple organs	?	0	21*	2/9	4	9.3	?	?	?
*Pegivirus K*	Porcine pegivirus	PPgV_903	KU351669	Pig	Serum	Pegi-like	0	0	4/4	2	9.1	?	Yes	?

?, Unknown or uncertain.

*Position of AUG initiation codon uncertain.

*Pegivirus A* includes GBV-A and other isolates from New World monkeys (U22303, U94421, AF023425 and AF023424) (Leary *et al.*, 1997; Simons *et al.*, 1995a) as well as viruses obtained from African bats (KC796085, KC796082, KC796086, KC796081, KC796075 and KC796089) (Quan *et al.*, 2013). *Pegivirus B* includes viruses (GBV-D) derived from bats in Asia (GU566735 and GU566734) (Epstein *et al.*, 2010) and Africa (KC796073 and KC796083) (Quan *et al.*, 2013). *Pegivirus C* is proposed as a new species to include GBV-C/hepatitis G virus (Leary *et al.*, 1996; Linnen *et al.*, 1996) and related viruses isolated from Old World primates (Bailey *et al.*, 2015; Birkenmeyer *et al.*, 1998; Kapusinszky *et al.*, 2015; Sibley *et al.*, 2014). Within this species, the virus phylogeny corresponds closely to that of the host (Bailey *et al.*, 2015; Sharp & Simmonds, 2011; Sibley *et al.*, 2014) with separate lineages for human (78 complete genome sequences), chimpanzee (AF070476), yellow baboon (KR996153, KR996142, KR996146, KR996144, KR996152, KR996151, KR996150, KR996149, KR996148, KR996147, KR996145, KR996143, KP890673 and KP890672), olive baboon (KF234530), red-tailed guenon (KF234529, KF234528, KF234526, KF234525 and KF234527), red colobus (KF234523, KF234524, KF234507,KF234522, KF234521, KF234520, KF234519, KF234518, KF234517, KF234516, KF234515, KF234514, KF234513, KF234512, KF234511, KF234510, KF234509, KF234508, KF234506, KF234505, KF234504, KF234503, KF234502, KF234501, KF234500 and KF234499) and African green monkey (KP296858). The proposed species *Pegivirus D* (KC145265) (Chandriani *et al.*, 2013) and *Pegivirus E* (KC410872) (Kapoor *et al.*, 2013b) both include single complete coding region sequences derived from horses. *Pegivirus F*, *G* and *I* all include viruses derived from Old and New World bats (Quan *et al.*, 2013), *Pegivirus H* includes viruses described as human pegivirus 2 and human hepegivirus (Berg *et al.*, 2015; Kapoor *et al.*, 2015), while *Pegivirus J* includes viruses derived from rodents (Firth *et al.*, 2014; Kapoor *et al.*, 2013a). *Pegivirus K* is a recently described virus isolated from pigs (Baechlein *et al.*, 2016). Some of the proposed species identifiers used will assist association with previous isolate names or designations (*Pegivirus A*: GBV-A, *Pegivirus C*: GBV-C, *Pegivirus E*: equine pegivirus and *Pegivirus H*: human pegivirus 2).

A division of the *Pegivirus* genus into two clades based on phylogenetic relationships (Kapoor *et al.*, 2015) could also be observed in our analyses [*Pegivirus A*, *B*, *C*, *D*, *E*, *I* and *K* (clade 1) and *Pegivirus* *F*, *G*, *H* and *J* (clade 2)]. Further investigation may support the suggestion that these clades differ from each other in internal ribosomal entry site (IRES) type; the reported correlation between these groupings with frequencies of *N*-linked glycosylation in the E1 and E2 proteins was not sustained (Table 2).

### Demarcation between genera

**Fig. 5.**
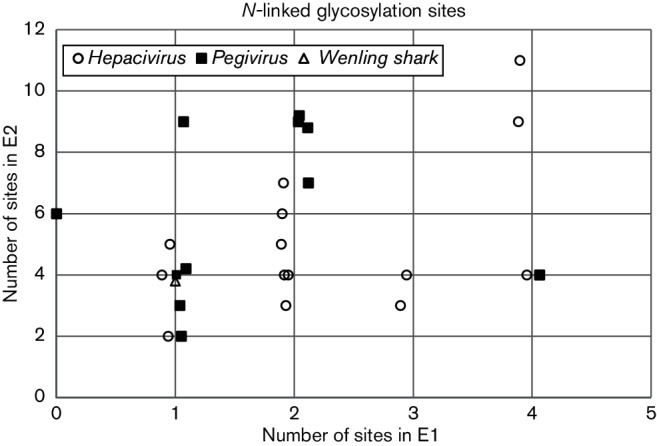
Predicted number of *N*-glycosylation sites. Scatter plots show the number of Asn-X-Ser/Thr glycosylation sites predicted for E1 (X-axis) or E2 (Y-axis) using NetNGlyc 1.0 Server (http://www.cbs.dtu.dk/services/NetNGlyc/) for individual species of *Pegivirus* (■), *Hepacivirus* (○) and for Wenling shark virus (∆). Points have been jittered on the X-axis to improve legibility.

Several characteristics have been used to differentiate members of the *Hepacivirus* and *Pegivirus* genera (Stapleton *et al.*, 2011). This expanded survey of diversity within these genera weakens some of these associations. For example, the number of *N*-linked glycosylation sites in the E1 and E2 glycoproteins was thought to be higher in members of the *Hepacivirus* genus, but with the expanded number of species considered here, this trend is no longer apparent either for E1 and E2 combined or when considered separately (Fig. 5). Similarly, a poly U tract of at least 10 residues is present in the 3′ UTR of some members of the *Hepacivirus* genus (Bukh *et al.*, 1999; Kolykhalov *et al.*, 1996; Scheel *et al.*, 2015; Tanaka *et al.*, 1995), but not others (Table 1). Some members of the *Hepacivirus* genus are hepatotropic and induce hepatitis, but for many species this information is unknown, while *Pegivirus D* (Theiler’s disease-associated virus) has been reported to be associated with serum hepatitis in horses (Chandriani *et al.*, 2013). Persistent infection can occur with members of either genus, but in many cases this information is lacking. The same difficulty applies to the characterization of virus IRES types; such regions are often lacking or incomplete despite the coding region being complete. In addition, in most cases no detailed molecular biology has been undertaken to confirm proposed secondary structures. However, even with these caveats, it is already clear that viruses with a similar type IV IRES (e.g. HCV) occur in members of both the *Hepacivirus* *and* *Pegivirus* genera (Tables 1, 2). The presence of ordered secondary structures across the genome as measured by mean free energy difference (MFED) values does not differ between the two genera.

**Fig. 6.**
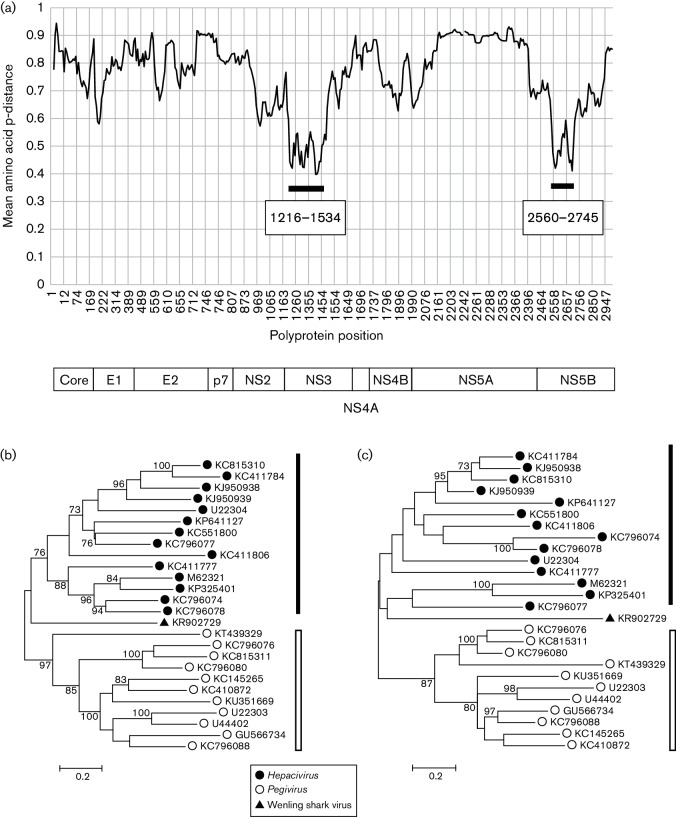
Comparative analysis of Wenling shark virus. (a) Mean amino acid p-distance across the virus polyprotein between single representatives of each *Hepacivirus* and *Pegivirus* species with Wenling shark virus was plotted against polyprotein position (numbered relative to M62321). The positions of two regions where mean p-distances were <0.6 are indicated by solid bars. A schema of the boundaries between virus proteins is given below. (b) Phylogenetic analysis of representatives of each *Hepacivirus* (filled circles) and *Pegivirus* (open circles) species was analysed together with Wenling shark virus (filled triangle) using amino acid sequences between positions (b) 1216–1534 and (c) 2560–2745. Trees were constructed from maximum-likelihood distances using a Le and Gascuel model with a gamma distribution of variation with a class of invariant sites. Branches supported by >70 % of bootstrap replicates are indicated.

Nevertheless, there remains a clear demarcation between the two genera in their phylogenetic relationships (Fig. 6). In addition, all members of the *Hepacivirus* genus have a long basic core region with between 20 and 34 lysine or arginine residues in the 156–216 residues between the presumed initiation codon and the presumed E1 cleavage site. Most members of the *Pegivirus* genus have a shorter and less basic sequence in this region or no identifiable coding sequence upstream of E1. However, a long and relatively basic regions is predicted in *Pegivirus J*.

### Wenling shark virus

Using the 14 *Hepacivirus* species and 11 *Pegivirus* species as references, we produced an amino acid alignment with the addition of Wenling shark virus (Shi *et al.*, 2015). The genome sequence possesses a predicted single ORF encoding 3087 amino acids, similar in length to polyproteins of hepaciviruses and pegiviruses. The 5′ UTR was short (131 bases) and potentially incomplete, but showed no identifiable regions of sequence homology with equivalent regions of hepaciviruses or pegiviruses. This suggests possible possession of an IRES type distinct from mammalian viruses. The 3′ end was 262 nucleotides in length, without the poly(U/C) tract observed in some hepaciviruses. The region could be predicted to possess RNA secondary structure with a series of stem-loops comparable to those of other members of the *Flaviviridae* family. The coding region of the genome possesses a predicted structured RNA with a mean MFED value of 18 %, higher than that of either hepaciviruses or pegiviruses.

The structural genes were predicted using the SignalP server to identify signalase cleavage sites characteristic of hepaciviruses and pegiviruses in this region, along with tentative alignment of cleavage sites between core/E1 and E1/E2 of hepaciviruses. This analysis predicts a relatively long core gene of 300 amino acids (nucleotide positions 132–1031), but with several regions of identifiable amino acid sequence homology towards the carboxy terminus of the protein. It also possesses 52 basic residues at the amino terminus consistent with an RNA packaging function. The predicted E1 protein (nucleotide positions 1032–1586, 185 amino acids) was similarly identifiably homologous to those of hepaciviruses with one predicted *N*-linked glycosylation site. The predicted E2 protein spans 272 amino acids (nucleotide positions 1587-2402) with four predicted *N*-linked sites, lower than for the E2 protein of most hepaciviruses.

Mean amino acid divergence between Wenling shark virus and either *Hepacivirus* or *Pegivirus* species was consistently less than 0.6 in two regions of the genome: between positions 1216 and 1534 and between 2560 and 2745 (numbered relative to M62321, Fig. 6). Phylogenetic analysis suggests that this virus is distinct from either genus, since each forms a separate clade in the region 1216–1534 (the *Hepacivirus* clade is supported by 68 % of bootstrap replicates), while for the region 2560–2745, the branch structure of the *Hepacivirus* clade, that includes Wenling shark virus, is poorly supported by bootstrap resampling. Overall, this analysis identifies a potentially greater overall similarity of Wenling shark virus to hepaciviruses, based both on the existence of predicted structural proteins homologous to those of hepaciviruses and the evidence for the grouping of Wenling shark virus with hepaciviruses on phylogenetic analysis of non-structural protein sequences between amino acid positions 2560–2745 (Fig. 6). However, without further information concerning the biology and molecular biology of this virus, we suggest it remains as an unclassified species within the *Flaviviridae*.

## Discussion

In recent years, there has been a remarkable increase in our knowledge of diversity within the *Hepacivirus* and *Pegivirus* genera. This change is largely due to the application of primer-independent deep-sequencing techniques to a wide range of mammalian host species. In 2011 there was evidence for two species in each genus (Stapleton *et al.*, 2011), whereas we now propose that the *Hepacivirus* genus should encompass 14 species (Table 1), while the *Pegivirus* genus should include 11 species (Table 2). The proposed criteria for demarcating between different species are divergence in two different regions of the virus genome. Very similar phylogenetic relationships have been reported for Bayesian or maximum-likelihood analysis based upon complete coding regions or subgenomic regions of subsets of these sequences (Baechlein *et al.*, 2015, 2016; Corman *et al.*, 2015; Kato *et al.*, 1990; Pfaender *et al.*, 2014; Quan *et al.*, 2013; Sibley *et al.*, 2014; Thézé *et al.*, 2015). We have chosen to base species demarcation criteria on amino acid p-distances for two defined regions of the virus genome (portions of the NS2/NS3 [protease] and NS5B [RNA-dependent RNA polymerase] proteins), since these regions can be easily aligned around conserved motifs with distances quickly computed using standard software.

In almost all cases, these distinctions are correlated with the known host range. One exception is *Hepacivirus A*, which was first described from dogs and described as canine hepacivirus (Kapoor *et al.*, 2011), but has since been reported at a high frequency in horses (Burbelo *et al.*, 2012; Kapoor *et al.*, 2013b; Lyons *et al.*, 2012; Postel *et al.*, 2015) and in only one subsequent report in dogs (El-Attar *et al.*, 2015). Phylogenetic analysis suggests that sequences derived from dogs are nested within those derived from horses, consistent with the detection of this virus in dogs being a secondary event, conceivably through the administration of vaccines manufactured using equine serum (Pybus & Thézé, 2015). The other species with a complex host range is *Pegivirus A* (GBV-A), which has been detected in New World primates and Old World bats. Virus variants within this species segregate with host species in the case of both primates (Bukh & Apgar, 1997) and bats (Quan *et al.*, 2013), consistent with a long period of co-evolution.

Our analysis does not include viruses represented only by partial genome sequences, although it is likely that these viruses include potential species within the *Hepacivirus* and *Pegivirus* genera additional to our proposed classification scheme (Drexler *et al.*, 2013; Quan *et al.*, 2013). In particular, we note that a large, diverse clade of bat-derived pegiviruses described from partial genome sequences (Quan *et al.*, 2013) is therefore excluded from our proposals. The reason for this decision is that, even though our phylogenetic analysis is based on subgenomic sequences, we do not feel that it is appropriate to propose species names for viruses for which the complete coding sequence remains uncharacterized and for which genome organization is only partly determined. The technical challenge of obtaining complete genome sequences of viruses, even without prior isolation, is considerably reduced following the advent of next-generation sequencing and, we believe, now justifies this requirement. Indeed, without this information, some of the important characters such as the presence of a basic core protein and the number of *N*-linked glycosylation sites may be unavailable and this might reduce the confidence of taxonomic assignments. Another reason for excluding subgenomic coding sequences from taxonomic proposals is that there is evidence for recombination between different species of *Hepacivirus* (Thézé *et al.*, 2015), this could not be properly assessed on subgenomic sequences. We have been less stringent with regard to the presence of non-coding regions of the genome since, although these contain features relevant to virus taxonomy, difficulties are sometimes experienced in sequencing these terminal regions of the genome. An example of the difficulties that could be produced by relying on subgenomic coding region sequences is provided by *Hepacivirus E* and *Hepacivirus F*, which might have been classified as a single species if comparisons had been limited to the region 2536–2959 (Fig. 2).

A similar argument could be made in terms of our incomplete knowledge of the biological and molecular properties (persistence, tissue tropism and pathogenicity) of viruses for which all that is known is their presumed primary host and complete genome sequence (Tables 1, 2). However, such additional information can be labour intensive and difficult to obtain, especially in viruses initially identified in wild fauna; in many cases this information may never be obtained. The requirement for a complete genome sequence at least makes it possible for future biological studies to be performed through assembly of synthetic infectious clones, as has been reported for *Hepacivirus A*, *Hepacivirus B*, *Hepacivirus C* (Bukh *et al.*, 1999; Kolykhalov *et al.*, 1997; Scheel *et al.*, 2015; Yanagi *et al.*, 1997) and *Pegivirus C* (Xiang *et al.*, 2000).

We have applied a more stringent test for Wenling shark virus, since although a complete coding sequence has been obtained and the host is known, phylogenetic analysis does not place this virus clearly within either the *Hepacivirus* or *Pegivirus* genera, although the arrangement of structural genes is most consistent with its eventual assignment as a *Hepacivirus*. Until biological and molecular information is obtained about this and other hepaci- or pegi-like viruses from non-mammalian hosts, we believe it is most prudent that this virus remains unassigned to either an existing or novel genus.

This survey of diversity within the *Hepacivirus* and *Pegivirus* genera somewhat obscures the demarcation criteria proposed to differentiate these genera (Stapleton *et al.*, 2011). Considerable overlap was observed between genera in 5′ UTR IRES type, the frequency of *N*-linked glycosylation (Fig. 5), the presence of poly U in the 3′ UTR, the persistence of infection and liver tropism (Tables 1, 2). At present the demarcation between these genera relies on the results of phylogenetic analysis (Fig. 6), the presence of one or more miR-122 sites (CACUCC) in the 5′ UTR followed by a basic core region, hepatotropism and liver pathology. We note that similar obscuration of demarcation criteria is likely to arise within these genera as additional complete genome sequences are obtained from a widening sample of host species by next-generation sequencing technology. It would be a mistake to regard such an outcome as a deficiency in current methods of taxonomy; genera and species are man-made categories imposed by us on a diverse virus fauna as a tool to organizing information and should be judged as such.

The evolutionary history of the *Hepacivirus* and *Pegivirus* genera is obviously complex and has been associated with multiple shifts of host species and genome mosaicism in the case of IRES sequences. Notable species-specific associations amongst primates are observed for *Pegivirus A* (Bukh & Apgar, 1997) and *Pegivirus C* (Sharp & Simmonds, 2011; Sibley *et al.*, 2014), consistent with co-speciation, whereas the presence of multiple *Pegivirus* species in bats and of multiple *Hepacivirus* species in rodents, as well as the relatedness of bat and primate-derived *Pegivirus* *A* isolates, is not. The primarily sequence-based species assignments proposed in the current study nevertheless divide the two genera into groups of viruses with a number of shared biological properties, either demonstrated or inferred, that will be of value in the future epidemiological, clinical and virological characterization of these viruses.

## Methods

Nucleotide sequences (other than HCV) assigned to the *Hepacivirus* genus and >6000 nucleotides in length were retrieved from GenBank and, together with single representatives of HCV genotypes 1–7, were aligned in their coding regions using muscle (Edgar, 2004) as implemented in sse v1.2 (Simmonds, 2012). Sequences differing by amino acid p-distances of <0.1 were then removed; this is a conservative cut-off since genotypes 1–7 of HCV differ by amino acid p-distances of 0.23–0.31. The final alignment consisted of sequences with the GenBank accession numbers M62321, D00944, D17763, Y11604, Y13184, Y12083, EF108306, KC411784, KC815310, KJ950939, KJ950938, KC411806, KC411777, KP325401, KP641127, KC551800, KC796078, KC796074, KC796077 and U22304. Scans of mean amino acid p-distances between groups of sequences were performed in sse (window size 50 amino acid residues, shifted by 10). Regions where mean amino acid p-distances were consistently <0.6 were adjusted visually to remove terminal poorly unaligned regions, and then used to produce histograms of amino acid distances. The optimal amino acid substitution model for each conserved region was assessed using mega6 (Tamura *et al.*, 2013) and used to produce maximum-likelihood trees. Frequency histograms were based on the p-distance between pairs of sequences.

The same process was carried out to produce an alignment of 160 genome sequences representing different *Pegivirus* variants. The final alignment, after removal of sequences differing by amino acid p-distances of less than 0.11, consisted of GenBank accession numbers U22303, U44402, AF070476, KR996153, KP296858, KF234529, KF234523, U94421, AF023425, AF023424, KT439329, KC410872, KC145265, KJ950934, KC815311, KC796085, KC796082, KC796086, KC796081, KC796088, GU566734, KC796073, KC796083, KC796087, KC796093, KC796076 and KC796084; we also included KU351669, a novel pegivirus recently isolated from a pig (Baechlein *et al.*, 2016). Separate analyses included the *Hepacivirus* and *Pegivirus* sets together with Wenling shark virus (KR902729).

*N*-linked glycosylation sites were predicted by analysis of envelope E1 and E2 fragments from examples of each species using NetNGlyc 1.0 (http://www.cbs.dtu.dk/services/NetNGlyc/). Cleavage sites in structural protein regions were independently predicted using the SignalP 4.1 server (http://www.cbs.dtu.dk/services/SignalP/). MFED values were calculated by comparing folding energies of consecutive fragments of nucleotide sequence to random sequence order controls using the program Folding Energy Scan in the sse package (Simmonds, 2012). Values represent the percentage difference between the MFE of the native sequence from that of the mean value of 50 sequence order randomized controls.
